# Transcription Factor T-Bet in Atlantic Salmon: Characterization and Gene Expression in Mucosal Tissues during *Aeromonas Salmonicida* Infection

**DOI:** 10.3389/fimmu.2015.00345

**Published:** 2015-07-06

**Authors:** Jaya Kumari, Zuobing Zhang, Trilochan Swain, Heng Chi, Cuijuan Niu, Jarl Bøgwald, Roy Ambli Dalmo

**Affiliations:** ^1^Faculty of Biosciences, Fisheries and Economics, Norwegian College of Fishery Science, University of Tromsø, Tromsø, Norway; ^2^Nofima, Tromsø, Norway; ^3^Ministry of Education Key Laboratory of Biodiversity Science and Ecological Engineering, College of Life Sciences, Beijing Normal University, Beijing, China; ^4^Key Laboratory of Experimental Marine Biology, Institute of Oceanology, Chinese Academy of Sciences, Qingdao, China

**Keywords:** Atlantic salmon, T-bet, *A. salmonicida*, gene expression, mucosal immunity

## Abstract

The T-box transcription factor T-bet is expressed in a number of hematopoietic cell types in mammals and plays an essential role in the lineage determination of Th1 T-helper cells and is considered as an essential feature for both innate and adaptive immune responses in higher vertebrates. In the present study, we have identified and characterized the full-length Atlantic salmon T-bet cDNA (3502 bp). The putative primary structure of the polypeptide deduced from the cDNA sequence contained 612 aa, which possessed a T-box DNA binding domain. Phylogenetic study and gene synteny revealed it is as a homolog to mammalian T-bet. Quantitative PCR analysis of different tissues in healthy fish showed that salmon T-bet gene was highly expressed in spleen, followed by head kidney, and was expressed in intestine, skin, and liver at lower levels. Moreover, the time-dependent expression profile of T-bet, interferon gamma (IFNγ), interleukin-22 (IL-22), and natural killer enhancement factor in mucosal tissues during water-borne infection with live *Aeromonas salmonicida*, indicated the involvement of T-bet in mucosal immune response in Atlantic salmon.

## Introduction

Lineage-restricted transcription factors are responsible for establishing the changing gene expression patterns that are required for the appropriate differentiation and functioning of each unique cell type of the body. Precisely, establishing these gene expression networks during development and in response to environmental stimuli is absolutely critical for maintaining cellular identity and functional capability.

The T-box transcription factor family is a key regulator of the cascade of gene expression events required for cellular specification during development (1). The original T-box family members were identified due to their critical role in embryonic development. The T-box family, defined by a common DNA binding domain known as T-box, is evolutionary ancient and probably arose in the common ancestor of metazoan organisms. The T-box gene family in mammals consists of 17 genes organized into 5 subfamilies (1). The importance of the T-box family in hematopoietic cell development has been recognized with the discovery of T-box expressed in T cells (T-bet), particularly in CD4^+^ T-helper 1 (Th1) cells and the subsequent identification of the overlapping expression profile of Eomesodermin (Eomes) in CD8^+^ T cells (2, 3). Two T-box genes of the Tbr1 subfamily, Eomes and T-bet (encoded by *Tbx21*), are important in the differentiation of T and B lymphocytes (4) as well as required for the generation of type 1 immunity in almost all the main cell types involved in both adaptive and innate immunity (5). Furthermore, T-bet has a unique role in the differentiation of all the three subsets of helper T cells by promoting Th1 differentiation, while simultaneously inhibiting the Th2 and Th17 lineage commitment programs (6).

However, in mammals, T-bet is also expressed in several different cell lineages of the hematopoietic system including DCs, natural killer (NK) cells, NKT cells, B cells, and CD8^+^ T cells, and several recent reports indicate that T-bet is also important in the lineage commitment of innate lymphoid cells (ILCs), and CD8αα intra-epithelial lymphocytes (IELs) similar to the conventional Th subsets (7). Moreover, in mammals, T-bet promotes the differentiation of ILCs in the gut, specifically the NKp46^+^ IL-7Ra^+^ innate lymphoid cell 1 (ILC1) and NKp46^+^CCR6^–^RORγt^+^ (NK-22 cells) ILC3 subsets (8–10).

It is acknowledged that the term “innate lymphoid cell” should be used to encompass the LIN− ILCs (lineage marker-negative ILCs), NK cells, and lymphoid tissue-inducer (LTi) cells. Furthermore, it has been suggested that ILCs be further divided into three subsets – group 1 ILCs (comprising ILC1s and NK cells), group 2 ILCs (comprising all innate cells producing type 2 cytokines), and group 3 ILCs (comprising ILC3s and LTi cells) based on their ability to produce type 1, type 2, and Th17 cell-associated cytokines, respectively. A recent report also has indicated a central role for T-bet in ILC3 development (11). Moreover, it is known that key transcription factors required for LTi development (Ikaros, Tox, RORγt, and Id2) have zebrafish orthologs, and associated genes show synteny between the human and zebrafish genomes (12). Teleost genomes also harbor orthologs of the transcription factor aryl hydrocarbon receptor (AHR), which is required for the expression of IL-22, and IL-22 is also found in zebrafish genomes that are clustered with interferon gamma (IFNγ) and interleukin-26 (IL-26) – as it is in higher mammals. Therefore, the genes essential for the generation of IL-22-expressing LTi may be highly conserved (12).

The best-studied mucosal T cells in fish (sea bass, carp, trout, and salmon) are IELs where most of them are CD3-ε^+^/CD8-α^+^ and display TCRγδ. These cells have been supposed to have cytotoxic and/or regulatory function (13). In the intestine, the IEL population is quite abundant in both fish, birds, and mammals (14). The functional relevance of IELs compared to T-bet expression has not yet been addressed in fish.

Most importantly, based on these recent findings in humans and mice, T-bet has emerged as one of the key players governing the transcriptional control of mucosal immune responses. Its expression in different immune cells found at mucosal surfaces may involve a critical balance between permitting robust host immunity and limiting susceptibility to various diseases (15). Moreover, Powell et al. (16) also demonstrated that T-bet is a crucial gatekeeper of innate inflammatory pathways at the intestinal barrier surfaces, where it regulates the balance between mucosal homeostasis and inflammation.

In teleosts, the roles of transcription factors have been reported for hematopoiesis in zebrafish (17) and for B-cell function in catfish (*Ictalurus punctatus*) (18) and fugu (*Takifugu rubripes*) (19). Unlike mammals, we still do not know in detail the full extent of T-cell diversity and the nature of initial signals that determine the T-cell response pattern in telesots. However, in Atlantic salmon (*Salmo salar*), several Th1/Th2/Th17/Treg cytokine genes (20–22) and the transcription factors *gata3*, *foxp3*, and *eomes* (23–25) have been identified in recent years. Moreover, T-bet has been identified recently in fish species like crucian carp (*Carassius carassius*) (26), rainbow trout (*Oncorhynchus mykiss*) (27), zebrafish (*Danio rerio*) (28), and grass carp (*Ctenopharyngodon idella*) (29), but T-bet have not been identified in Atlantic salmon so far. In the present study, we isolated full-length cDNA of Atlantic salmon T-bet (Tbx21) and characterized it in terms of sequence alignment, gene synteny, and tissue expression patterns. We also aimed to identify a few mediators that control the expression of T-bet in fish lymphocytes using quantitative PCR. Since, T cells and ILCs play an essential role in regulating mucosal immune responses in the gastrointestinal tract and mucosal surfaces in higher vertebrates and as yet there is no evidence of T-bet being involved in mucosal immune response in teleost, we proceeded to investigate the role of T-bet in mucosal tissues in Atlantic salmon. The main focus in the present study was to examine the crosstalk between T-bet and associated genes at the transcript level using water-borne *Aeromonas salmonicida* infection model.

## Materials and Methods

### Fish

Atlantic salmon weighing 70–100 g were kept at the Aquaculture Research Station (Tromsø, Norway) in circular 200 l tanks supplied with freshwater at an ambient temperature of ~10°C with 12/12 h illumination, and the fish were fed a commercial pelleted diet. Prior to treatment or challenge, fish were anesthetized in 0.005% benzocaine. Fish were sacrificed using 0.01% benzocaine prior to collection of different tissues. The challenged fish were also kept at the same conditions as before challenge. The experimental protocols used for Atlantic salmon in this study were reviewed and approved by the Committee on the Ethics and Animal Welfare of Norway, the National Animal Research Authority (NARA)/(Norwegian: Forsøksdyrutvalget, FDU), http://www.fdu.no.

### Molecular cloning and sequencing of atlantic salmon (As) T-bet cDNA

Using a BLAST search against crucian carp T-bet protein sequence and against nucleotide collection library in GenBank^1^ , we identified a partial cDNA sequence of a T-bet homolog, predicted within a salmon genomic sequence (GeneBank Accession No. EU025708.1). The deduced protein sequence (Acc No. ABW77500.1) had previously been denoted as salmon T-box transcription factor eomesodermin. Internal primers were designed from the known sequence and AsT-bet clones were obtained from the cDNA library obtained from the stimulated spleen tissue and sequenced. 3′ and 5′ RACE were performed using a GeneRacer™ Kit (Invitrogen, Carlsbad, CA, USA) according to manufacturer’s instruction. Total RNA (3 μg) isolated from Atlantic salmon spleen (~80 mg) tissue using TRIZOL^®^ Reagent (Invitrogen) was used as a template and reverse transcribed using Superscript™ III RT and the GeneRacer Oligo dT primer. Primers used for the 5′ or 3′ RACE are listed in Table 1. PCR products were gel purified using MinElute Gel Extraction kit (QIAgen, Hilden, Germany) and cloned in a TOPO vector (Invitrogen). Plasmid DNA from at least 10 independent clones was purified using QIAprep Spin Miniprep kit and sequenced. The cDNA sequence and deduced amino acid sequence of Atlantic salmon T-bet were analyzed using the BLAST program, the ExPASy Molecular Biology server^2^ and Pfam. Amino acid identity and similarity were done with the Matrix Global Alignment Tool (MatGAT) program v 2.0 (30) using default parameters. Multiple amino acid sequence alignments were constructed with ClustalW2 program and further edited using GeneDoc, version 2.7. Phylogenetic tree was constructed using the neighbor-joining (N-J) method using the MEGA v 4.0 program (31). The reliability of the branching topology was tested by bootstrap re-sampling (10,000 pseudo-replicates).

**Table 1:** **List of primers and their designated applications**.

Primer name	Sequence (5′–3′)	Application	GenBank accession number
AsTbet-F	CCATCGCTGCAGGACAAGCCCAAG	3′RACE/cDNA cloning	
AsTbet-NestF	CGGTCAAATCGGTGGACTCTGCTG	3′RACE	
AsTbet-R	CGCCCTCAAACAGGCCGGAGTC	5′RACE/cDNA cloning	
AsTbet-NestR	CCTTGGGCTTGTCCTGCAGCGATG	5′RACE	
AsTbet-F	GTACCACGCAGACCAAACTG	5′RACE extension	
AsTbet-R	GGGCAGAGGGTTCATGTAC	5′RACE extension	
M13 F	CAGGAAACAGCTATGAC	Sequencing	
M13 R	GTAAAACGACGGCCAG	Sequencing	
T3 F	ATTAACCCTCACTAAAGGGA	Sequencing	
T7 R	TAATACGACTCACTATAGGG	Sequencing	
AsTbet-F	GGCATAGGTGGCAATCTTTACC	Real-time PCR	GU979861
AsTbet-R2	GTGCCGATCCGCCCTGTC	Real-time PCR	
AsNKEF-F	TGCCGAGGAGTTTAGGAAGA	Real-time PCR	NM_001141386
AsNKEF-R	AATCTTCATGGCACCCAGAC	Real-time PCR	
AsIL22-F	GGCCCGAGTCAGCAGAGACCT	Real-time PCR	DW572073
AsIL22-R	CTCCTCCATCCCGGCCAACTTC	Real-time PCR	
AsIFNγ-F	CGTGTATCGGAGTATCTTCAACCA	Real-time PCR	NM_001123558
AsIFNγ-R	CTCCTGAACCTTCCCCTTGAC	Real-time PCR	
AsEFα-F	CGGCAAGTCCACCACCAC	Real-time PCR	AF308735
AsEFα-R	GTAGTACCTGCCAGTCTCAAAC	Real-time PCR	

### Gene synteny analysis

T-bet gene orthologs, in fugu, tetraodon (*Tetraodon nigroviridis*), medaka (*Oryzias latipes*), zebrafish (*D. rerio*), mouse (*Mus musculus*), and human (*Homo sapiens*), were identified in the Ensembl genome database^3^, and their locations in genomic loci were compared with salmon’s (Acc No. EU025708.1) (32).

### Tissue specific expression of AsT-bet by real-time PCR (qPCR)

The expression patterns of T-bet in different tissues of healthy fish were measured by real-time PCR. From healthy fish, tissues from different organs, such as liver, spleen, head kidney, gill, thymus, intestine, heart, skin, brain, ovary, and testis, were collected in RNA-later (Ambion, Austin, TX, USA) and subsequently processed for RNA isolation. Total RNA was extracted with TRIZOL reagent and purified using the PureLink™ RNA Mini Kit (Life Technologies). Purified RNA was confirmed to be intact by gel electrophoresis, while RNA concentration and purity were measured spectrophotometrically (Nano-Drop Technologies, Wilmington, DE, USA). To remove any contaminating genomic DNA, samples were treated with DNase I, amplification grade (1 U μg^−1^ RNA; Invitrogen). The SuperScript III RNase H^−^ reverse transcriptase (Invitrogen) was used to synthesize first-strand cDNA with oligo(dT)18 primer from 1 μg of total RNA at 50°C for 50 min.

qPCR was performed in triplicates with an ABI PRISM 7500 Fast Real-Time PCR System (Applied Biosystem) using Fast SYBR^®^ Green (Applied Biosystem). Reaction mixtures were incubated for 10 min at 95°C, followed by 40 cycles of 15 s at 95°C, 1 min at 60°C, and, finally, 15 s at 95°C, 1 min 60°C, and 15 s at 95°C. In all cases, amplifications were specific and no amplification was observed in negative controls (non-template control and non-reverse transcriptase control). The Ct values for each sample were converted into fold differences according to the relative quantification method described by Pfaffl (33). EF-1α was found to be the most stable reference gene compared to 18S rRNA. So, gene expression was normalized by the EF-1α in each sample. The primers used are shown in Table 1.

### *In vivo* infection

*Aeromonas salmonicida* (Strain LFI 4017) has been proven to cause mortalities in previous and standardized experiments. In the cohabitation challenge experiment with *A. salmonicida*, fish were obtained from AquaGen (Sunndalsøra, Norway). Fifty fish (50–80 g) were placed in a 500 l tank and the fish were acclimatized for 1 week prior to challenge under the same experimental conditions mentioned in Section “Fish.” Six fish were infected with *A. salmonicida* (injected i.p with 1 × 10^8^ colony forming units of live bacteria in PBS, overnight culture) and placed in the tank. All habitants (shedders) died at day 6–8. Sampling was conducted 0, 5, 8, 10, 14, and 21 days after the shedders were added to the tank. Zero-day samples were controls. The fish started to die at day 14, about 1 week after the shedders died. After 41 days, ~15% of the fish were dead. The fish died from furunculosis as the bacterial subcultures made from kidney samples were verified as *A. salmonicida*, and no other bacterial species were detected after plating and inoculation from the head kidney samples. Skin, spleen, head kidney, and intestine were collected from four fishes at each time-point. All organs were rapidly transferred to and kept in RNA-later (Ambion, Austin, TX, USA) and subsequently processed for RNA isolation, cDNA, and qPCR as described in Section “Tissue Specific Expression of AsT-bet by Real-Time PCR (qPCR).”

### Primary cell culture and stimulation of spleen leukocytes

Three fish (1–1.5 kg) were anesthetized with benzocaine, the spleen was aseptically removed and placed in universal tubes containing Leibowitz medium (L-15) containing 100 U/ml penicillin, 100 μg/ml streptomycin, 0.5% FBS, and 40 U/ml heparin (referred herein as incomplete medium, L-15i). The tissue was gently pushed through sterile 100-μm mesh screens and the screens were rinsed with L-15i. The resulting cell suspension was layered on 25/54% Percoll gradient (GE Healthcare, Oslo, Norway) and centrifuged at 400 × *g* for 20 min at 4°C. Cells at the 25/54% density interface were collected, washed twice in L-15i by centrifugation at 400 × *g* for 10 min at 4°C, and the leukocytes were then re-suspended in complete medium (the same as incomplete medium but with 5% FBS) at 2 × 10^6^ cells/ml. One milliliter of cells were seeded in 12 well tissue culture plates and incubated at 14°C for 24, 48, and 72 h for each stimulant or combination of stimulants or no stimulants, i.e., only media control. The leukocytes were stimulated with known mitogens for T-cell stimulation, i.e., PHA (10 μg/ml) + ConA (10 μg/ml) + recombinant human IL-2 (rhIL-2) (1 ng/ml), and with recombinant Atlantic salmon IFN-α2 at two dose levels: 5 ng/ml and 0.5 μg/ml.

### Data analysis

The log-transformed data were analyzed by one-way analysis of variance (ANOVA) followed by Tukey’s test using SPSS 19.0 software. Differences were considered statistically significant when *P* < 0.05.

## Results

### Sequence analysis and characterization of salmon T-bet

**Figure 1:**
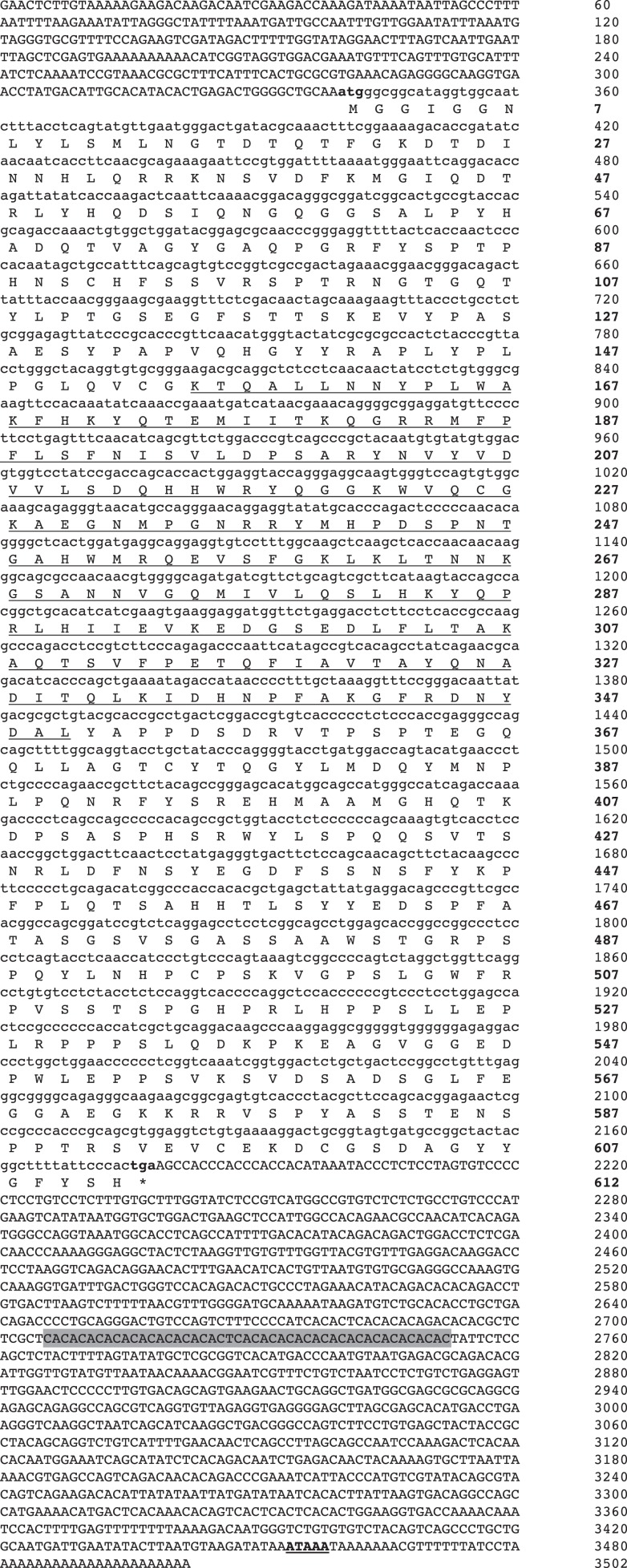
**Nucleotide and deduced amino acid sequence of Atlantic salmon T-bet cDNA**. Uppercase denotes the UTR’s and lowercase denotes the coding regions. The T-box DNA binding domain is underlined. Start and stop codons are marked with bold letters. The asterisk indicates the stop codon. The putative polyadenylation signal is bold and underlined. A region of repeat with 23 copies of the consensus sequences CA (2 bp) is shaded.

To obtain full-length cDNA of AsT-bet, 5′ and 3′ RACE were performed using spleen derived cDNA as template. The Atlantic salmon T-bet cDNA sequence (GenBank accession no. GU979861) consisted of 3502 bp in length including 339 bp 5′ untranslated region (UTR), a 1324 bp 3′-UTR and possessed an open reading frame of 1839 bp (Figure 1). In the 3′-UTR, there is a polyadenylation signal (AATAAA) 21 bp upstream of poly-A tail and in addition, one region of repeat with 23 copies of the consensus sequence CA (2 bp) was also identified in the 3′-UTR using tandem repeats finder program (34). The putative T-bet protein in Atlantic salmon was predicted to be 612 aa long, with a calculated molecular weight of 67.8 kDa and a pI of 8.02. The conserved T-box DNA binding domain consisted of 196 aa.

**Figure 2:**
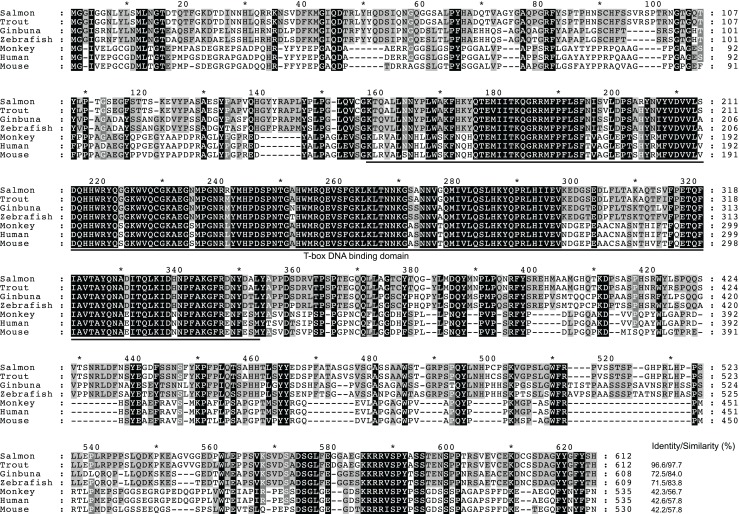
**Multiple sequence alignment of the deduced amino acid sequences from salmon T-bet and other vertebrates by the ClustalW2 program**. Residues shaded in black are completely conserved across all species aligned, and residues shaded in gray refer to 60–80% identity. Dashes indicate gaps. The T-box DNA binding domain is indicated by solid line below the alignment. Accession numbers are given in Figure 3.

**Figure 3:**
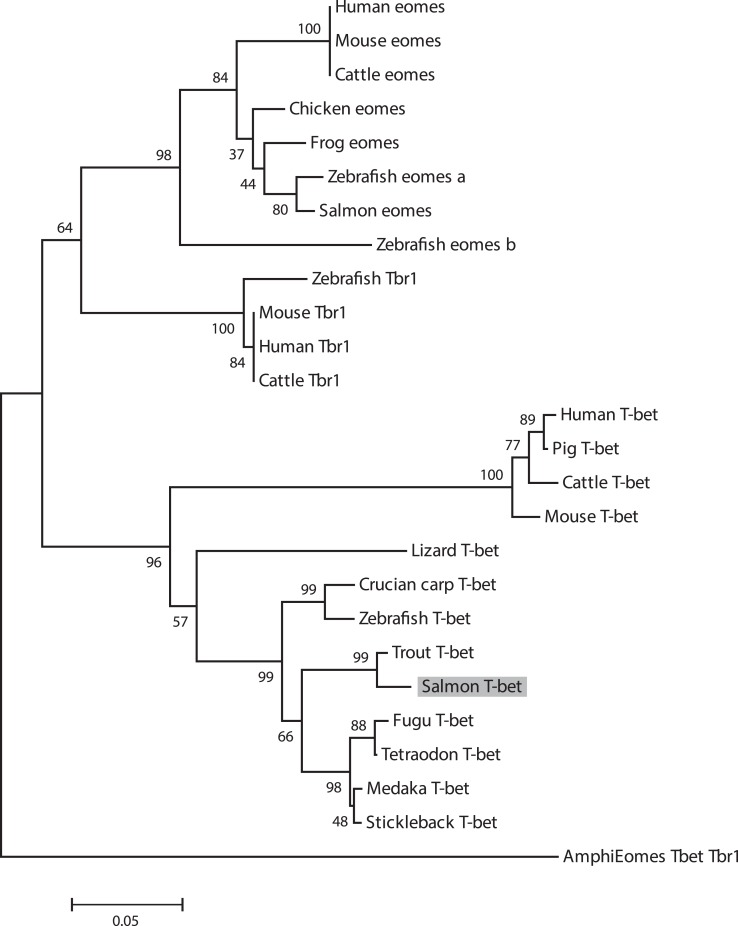
**Phylogenetic tree showing the relationship of salmon T-bet gene with other known vertebrate members of the Tbr1 subfamily**. The phylogram was constructed on ClustalX2 and MEGA 4.1. The neighbor-joining (N-J) method with bootstrap values of 1000 replications was adopted. Accession numbers or ENSEMBL gene IDs are as follows: Human (*Homo sapiens*) Eomes, NP_005433; Mouse (*Mus musculus*) Eomes, NP_034266; Cattle (*Bos taurus*) Eomes, NP_001178117; Frog (*Xenopus laevis*) Eomes, NP_001081810; Chicken (*Gallus gallus*), Eomes, XP_426003; Zebrafish (*Danio rerio*) Eomes a, NP_571754; Zebrafish Eomes b, NP_001077044; Salmon (*Salmo salar*) Eomes, EU418014; Amphioxus (*Branchiostoma floridae*) Eomes/Tbr1/Tbx21, AF262568; Human Tbr1, NP_006584; Mouse Tbr1, NP_033348.2; Cattle Tbr1, NP_001178978; Zebrafish Tbr1, NP_001108562; Human T-bet, NP_037483; Mouse T-bet, NP_062380; Cattle T-bet, NP_001179069; Pig (*Sus scrofa*) T-bet, ENSSSCP00000018565; Frog T-bet, NP_001088247; Lizard (*Anolis carolinensis*) T-bet, ENSACAP00000006911; Medaka (*Oryzias latipes*) T-bet, ENSORLP00000015259; Fugu (*Takifugu rubripes*) T-bet, ENSTRUP00000032964; Stickleback (*Gasterosteus aculeatus*) T-bet, ENSGACP00000005023; Tetraodon (*Tetraodon nigroviridis*) T-bet, ENSTNIP00000013001; Zebrafish T-bet, NP_001164070; Crucian carp (*Carassius auratus langsdorfii*) T-bet, BAF73805.1; Trout (*Oncorhynchus mykiss*) T-bet, NM_001182722.

**Figure 4:**
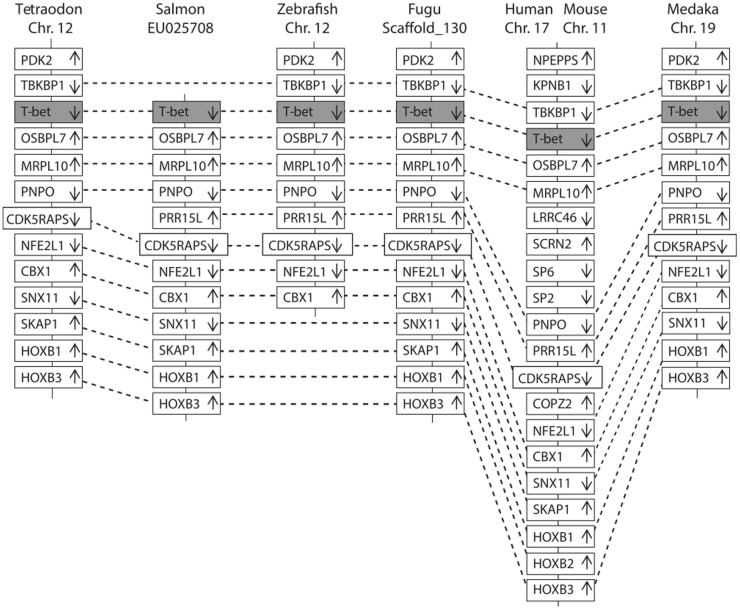
**Comparison of the location of T-bet genes in fish and mammals**. The chromosomes and scaffold were identified in ENSEMBL (http://www.ensembl.org/). Boxes represent the deduced genes. Arrows indicate the deduced orientation of the gene transcription. Dash lines connecting boxes suggest the homologous relationship.

In Figure 2, a multiple alignment of amino acid sequence in salmon and other vertebrates is depicted. Salmon T-bet shared 96.6, 72.5, and 42.2% amino acid identity with rainbow trout, zebrafish, and human T-bet, respectively (Table S1 in Supplementary Material). Compared to teleost, there were gaps in both in the 5′ and 3′ end of the amino acid sequence in mammals, monkey, and mouse – especially at the 3′ end side, resulting in shorter and divergent sequences compared to those in teleost. The phylogenetic study (Figure 3) showed that teleost T-bet formed an independent clade, but grouped with mammalian T-bet with a high bootstrap value (96%) that was higher than the other members of Tbr1 subfamily. This finding supported the notion that the salmon T-bet is a real T-bet ortholog. Furthermore, gene synteny analysis (Figure 4) revealed it is a homolog to mammalian T-bet having the same loci order and orientation: OSBPL7, MRPL10, PNPO, PRR15L (except in tetraodon), CD5KPRAP3, NFE2L1, and CBX. In addition, TBKBP1 in fugu, tetraodon, medaka, zebrafish, human, and mouse was observed to, with the same orientation, locate in the same position as T-bet gene in the corresponding genome.

### Tissue distribution of T-bet expression in healthy salmon

**Figure 5:**
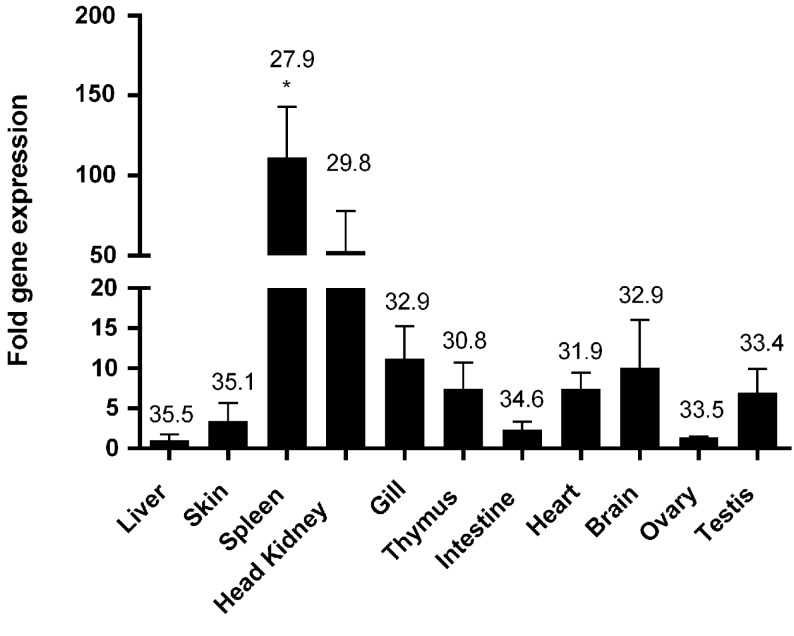
**Tissue distribution of T-bet expression in healthy Atlantic salmon**. Expression of salmon T-bet in different organs by real-time PCR. Gene expression data were normalized to EF-1α expression using liver as a calibrator. Bar represents the mean ± SEM (*n* = 6). Asterisk (*) above the bar shows significant difference (*P* < 0.05) compared with the organ that showed the lowest expression (liver). The value above the bars shows average real-time CT values of six fish.

Quantitative PCR analysis showed that salmon T-bet gene was ubiquitously expressed in all the tissues analyzed, but was highly expressed in the spleen and head kidney showing about 100 and 50 times higher expression than that of intestine, skin, and ovary. Moderate transcript levels were observed in gill, brain, thymus, and testis, whereas weak expression was detected in the intestine, ovary, and skin with the lowest expression in the liver (Figure 5).

### *In vitro* modulation of T-bet expression in spleen leukocytes

**Figure 6:**
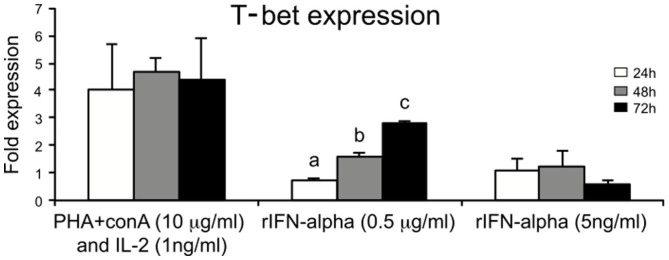
**Regulators of T-bet expression in salmon leukocytes**. Spleen leukocytes were stimulated for 24, 48, and 72 h with ConA + PHA + huIL-2, IFN-α (0.5 mg/ml and 5 ng/ml), and the mRNA levels of T-bet were determined by real-time PCR. Gene expression is normalized against EF-1α and is shown relative to the mean of the non-stimulated cells (leukocyte cells with no treatment, i.e., only media control). Spleen leukocytes with no treatment were considered as control for each time points. Each bar represents the mean ± SE of triplicate samples. Different letters denote statistically significant differences (*P* < 0.05) between the groups.

Some stimulants known to activate lymphocytes were tested for their potency to regulate the expression of T-bet in spleen leukocytes. Interestingly, stimulation of spleen leukocytes with known T cell stimulants, i.e., PHA + ConA and rhIL-2 resulted in the strongest induction of T-bet expression starting from 24 h and it was maintained until 72 h of incubation. Stimulation with recombinant salmon IFN-α2 (0.5 μg/ml) also showed gradual and significant higher expression of T-bet from 48 h and onward compared to expression at 24 h (Figure 6).

### *In vivo* regulation of salmon T-bet expression

To elucidate the mechanisms that regulates T-bet expression or whether T-bet might be co-regulated with other genes *in vivo* during mucosal and systemic infection, we utilized an experimental challenge model involving water-borne infection with *A. salmonicida*. We also tried to compare the mRNA expression levels of T-bet in mucosal and lymphoid tissues to highlight the characteristics of T-bet expression during the water-borne infection and to explore how the expression of T-bet might regulate other genes involved in Th1-like response. Therefore, we first examined the mRNA expression levels of AsT-bet, IFNγ, IL-22, and natural killer enhancement factor (NKEF)-A at different time intervals following bacterial infection in the lymphoid and mucosal tissues of the co-habitants, i.e., spleen, head kidney, skin, and intestine, respectively.

**Figure 7:**
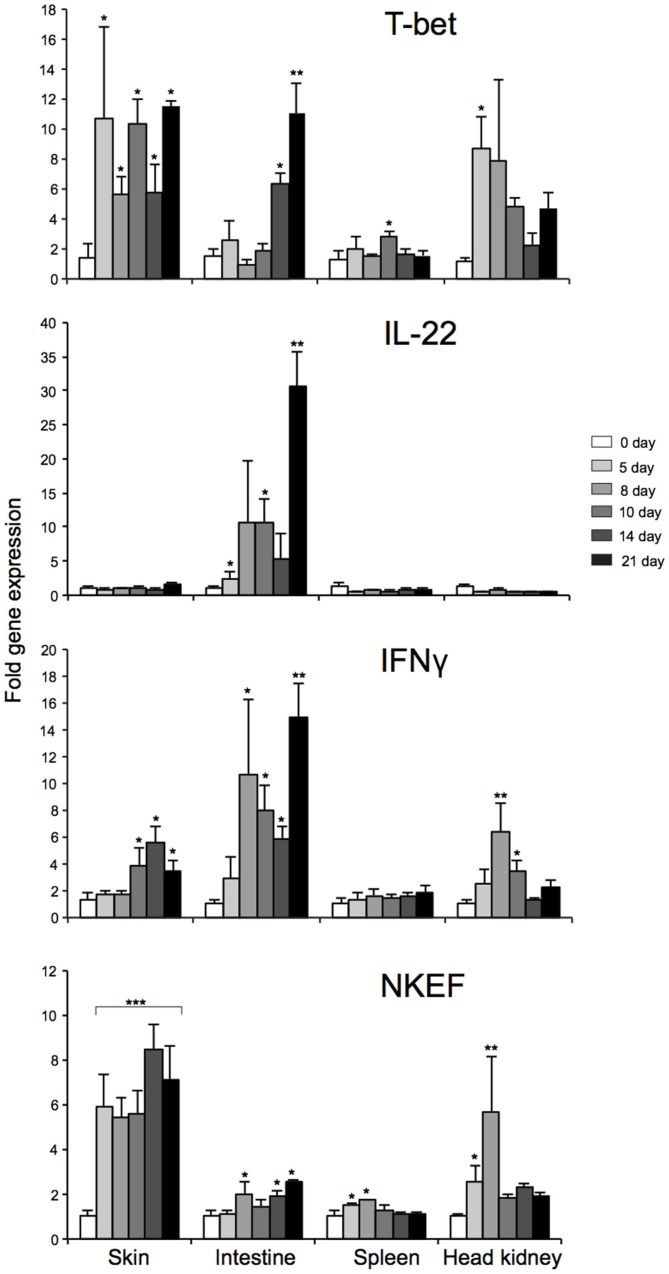
**Tissue specific expression of T-bet, IL-22, NKEF, and IFNγ in infected Atlantic salmon at different time-points after water-borne infection with *A. salmonicida***. Bars represent the relative expression levels of T-bet, IL-22, NKEF, and IFNγ normalized to EF-1α. Each value represents the mean ± SEM (*n* = 4). Statistical differences (*P* < 0.05, *P* < 0.01, and *P* < 0.001) between different time-points compared to control are indicated by asterisk (*,**, and ***) respectively, above the bars.

During water-borne infection with *A*. *salmonicida*, early (day 5) and strong T-bet expression in skin and head kidney was found (*P* < 0.05), whereas a moderate and delayed expression (*P* < 0.05) in the spleen (at day 10) and moderate to strong and also delayed expression (*P* < 0.05, 0.01) in the intestine (day 14 and 21) were observed, respectively (Figure 7). Similarly, NKEF expression was also significantly upregulated in all the tissues, especially in skin (*P* < 0.001) at all the time-points post challenge. This noticeable up-regulation of T-bet and NKEF at all the time-points in skin, points to an interesting co-regulation. Furthermore, IFNγ expression was, in general, also significantly upregulated in all the tissues of the co-habitants after 8–10 days of challenge except in the spleen. Alternatively, IL-22 showed a different response of strong significant expression only in intestine, suggesting the presence of ILC3-like subset that specifically secretes IL-22 during the response (may be T-bet regulated), similar to mammals. In spleen, head kidney, and skin, IL-22 expression level was similar to that of the control fish.

To sum up, the overall expression patterns in different tissues, T-bet, NKEF, and IFNγ showed similar profile of overall expression. On the other hand, IL-22, IFNγ, T-bet, and NKEF showed similar pattern of expression with a peak at 21 days post challenge, particularly in the intestine, thus, indicating a strong correlation between them.

## Discussion

T-bet has emerged as one of the key transcription factor responsible for controlling the fate of both innate and adaptive immune cells and its activity in different immune cells found at the mucosal surfaces is capable of dictating the critical balance between permitting robust host immunity and limiting susceptibility to disease in mammals (15).

In this study, we have cloned the full-length cDNA of T-bet from Atlantic salmon. We have proposed the presence of T-bet in Atlantic salmon that possesses high degree of partial amino acid homology to T-bet from other animal species. Especially, the deduced amino acid sequence showed overall highest identity (96.6%) with rainbow trout, but a limited overall amino acid identities to mammalian T-bets (42–43%) due to the divergent N and C terminal regions. Atlantic salmon T-bet contains a T-box binding domain that is a typical feature of T-box family genes and is highly conserved among all the T-bet proteins from fish and mammals (Figure 2). Phylogenetic analysis, gene organization, and conserved synteny indicated that AsT-bet is an ortholog of mammalian T-bet, which is in accordance with the synteny analysis in crucian carp performed by Takizawa et al. (26). We have also examined the expression of AsT-bet *in vivo* in healthy fish and *in vitro* in spleen leukocytes modulated by different stimulants. The normal tissue expression in healthy fish showed that AsT-bet was widely expressed in most of the tissues and strongly expressed in spleen and head kidney, which is in agreement with the previous reports on expression of T-bet in crucian carp (26), rainbow trout (27), zebrafish (28), and grass carp (29).

To delineate the mechanisms that regulate T-bet expression *in vitro*, this study identified IFN-α, a known antiviral component (35–37) and known T-cell stimulators as an important inducer of T-bet expression, which may in turn regulate the differentiation of Th1, NK, NKT, ILCs, and IELs. Similar studies done in rainbow trout also showed upregulated expression of T-bet in splenocytes after PHA treatment (27). In the present study, we assumed that the stimulatory effect of T-bet expression on splenocytes was either due to mitogens alone (PHA + ConA) or may be due to symbiotic effect of mitogens and rhIL-2. Since the effect of rhIL-2 was not studied separately, we cannot give any proof whether there was any effect of rhIL-2. In general, the use of, e.g., cytokines from other animal species than the study species should be avoided. In addition to the antiviral effects, IFN-α exerts pleiotropic cellular effects, such as inhibition of cellular proliferation, induction of apoptosis, and differentiation as well as modulation of the immune system. The latter effect includes inhibition of T-lymphocyte activation, enhancement of the cytotoxic activity of NK cells and T lymphocytes (38). Furthermore, ILCs are an emerging family of effector cells that contribute to lymphoid organogenesis, metabolism, tissue remodeling, and protection against infections. They maintain homeostatic immunity at barrier surfaces, such as lung, skin, and gut in mammals (39). In the present study, we tried to delineate the role of T-bet in salmon immune response and also in the context of mucosal immunity targeting the barrier surfaces like the skin and intestine. Our result shed new light on the T-bet expression in correlation with other candidate genes that tickled us speculating whether ILCs (NK-like cells and LTi-like cells) are present in teleosts.

Natural killer enhancement factor, which have been sequenced from several fish species [reviewed by Chen et. al. (40)] and have been reportedly expressed in all major fish organs, including the major mucosal surfaces (41), indicates its triggering effect on NK-like cells in teleost as described for mammals during infection. Earlier reports have revealed relationship between fish NKEF mRNA expression and bacterial or viral infection (40, 42). Some studies have shown a higher mRNA expression of NKEF in spleen and head kidney of turbot after challenge with *Vibrio anguillarum*, as well as in rainbow trout during viral hemorrhagic septicemia (VHS) (43). In another study in Atlantic salmon infected with *Caligus* sp., presence of NKEF like protein in gills and skin were observed (44). The present study is in accordance to the previous report, showing the enhancement of NKEF in skin, intestine, spleen, and head kidney, and this enhancement could be related to the activation of NK-like cells (group 1-like ILCs) in the lymphoid and mucosal tissues studied in response to *A. salmonicida* infection. Furthermore, our findings showed robust or more rapid significant up-regulation of NKEF and T-bet followed by IFN-γ expression/transcript, especially in the skin, highlighting the possibility, as discussed above, of group 1 ILC-like population including NK-like cells at the skin barrier in salmon, which may in turn have evolved to respond immediately to pathogenic signals and to aid the adaptive immune system in mounting a robust T-bet mediated type 1 immune response against bacterial infection.

IL-22 is known to be involved in innate defense against extracellular pathogens and microbes. A recent study revealed that a new subpopulation of mucosal NK-like cells secrete IL-22, particularly in the intestine. In addition, a different subset of NK cells, named NK-22, has been shown to be specialized in IL-22 production in mucosal-associated lymphoid tissue in mouse and human providing mucosal protection (45). In teleost, IL-22 homologs have been identified in several species including rainbow trout (45). Studies in rainbow trout have shown that IL-22 is highly expressed in intestine, suggesting a potential role in mucosal immunity (45). They also summarized that trout IL-22 enhance innate immune response by triggering antimicrobial defenses to facilitate microbial clearance similar to mammals. Moreover, it has been proposed that the gastrointestinal tract is the principal infection route of *A. salmonicida* in various salmonid species, such as Arctic charr (*Salvelinus alpinus*), Atlantic salmon, and rainbow trout (46). Similarly, in the present study, high expression of IL-22, particularly in the intestine during post challenge with *A. salmonicida* may also point to an immunological role of IL-22 in the intestine during bacterial infection. Recent studies in trout showed that IELs do respond strongly following an *A. salmonicida* infection – showing its important role in *A. salmonicida* protection (47). Concurrently, expression profiles of T-bet, IFN-γ, and IL-22 in this study, might suggest the positive correlation of T-bet with IL-22 expressing cells in the intestine during *A. salmonicida* infection. As described before, the genes essential for the generation of IL-22-expressing LTi are likely to be present in teleost species. These results might suggest that LTi (which are regarded as group 3 ILCs in mammals) mediated IL-22 expression is vital for intestinal homeostasis and innate immune response to extracellular bacteria. This shows that group 3 ILCs may be present in teleost, but further studies will be needed. On the other hand, we can also speculate on specific T-bet mediated control of IL-22 secreting NK cells and/or IL-22-expressing LTi-like cells, mucosal lymphoid cells (IELs) in the intestine.

To conclude, cloning and characterization of the salmon T-bet gene following with *in vitro* and *in vivo* experiments have provided new insight into the immune response of T-bet gene at the mucosal sites in fish. From the present findings, it is conceivable to speculate that T-bet together with IL-22, NKEF, and IFNγ might have a central role in the mucosal immune response to *A. salmonicida* infection in Atlantic salmon. The observation highlights the requirement for a more detailed characterization of T-bet in mucosal lymphoid cells together with functional studies.

## Conflict of Interest Statement

The authors declare that the research was conducted in the absence of any commercial or financial relationships that could be construed as a potential conflict of interest.

## Supplementary Material

The Supplementary Material for this article can be found online at http://journal.frontiersin.org/article/10.3389/fimmu.2015.00345

## Footnotes

^1^http://www.ncbi.nlm.nih.gov/BLAST

^2^http://us.expasy.org

^3^http://www.ensembl.org/index.html
