# Supplementary material for: Transcription Factor T-Bet in Atlantic Salmon: Characterization and Gene Expression in Mucosal Tissues during Aeromonas Salmonicida Infection
Source: Front Immunol. 2015 Jul 6;6:345. doi: 10.3389/fimmu.2015.00345 (PMC4492157; doi:10.3389/fimmu.2015.00345)
Supplement: Supplementary file 1 [file table_1.docx]

**Supplementary Table S1 - Similarity and identity of T-bet in some vertebrates at amino acid level.** The values of similarities are in columns and identities are in rows and shaded. The highest and the lowest are highlighted in bold. Matrix Global Alignment Tool (MatGAT v2.01) was applied in the measure.

|  | 1 | 2 | 3 | 4 | 5 | 6 | 7 |
| --- | --- | --- | --- | --- | --- | --- | --- |
| - Salmon |  | **96.6** | 72.5 | 71.5 | 42.3 | 42.6 | **42.2** |
| - Trout | **97.7** |  | 72.0 | 71.2 | 42.1 | 42.5 | 42.4 |
| - Ginbuna | 84.0 | 84.0 |  | 91.0 | 43.5 | 43.1 | 42.2 |
| - Zebrafish | 83.8 | 83.8 | 95.4 |  | 43.5 | 44.2 | 43.8 |
| - Mouse | 56.7 | 57.4 | 58.6 | 57.6 |  | 86.5 | 86.9 |
| - Monkey | 57.8 | 57.0 | 57.4 | 57.6 | 91.4 |  | 97.9 |
| - Human | **57.8** | 56.9 | 57.4 | 57.3 | 91.8 | 97.9 |  |
